# Economic impact of initial glaucoma treatment with selective laser trabeculoplasty on the Brazilian Public Health System

**DOI:** 10.5935/0004-2749.2024-0215

**Published:** 2024-12-26

**Authors:** Ivan M. Tavares, Flavio E. Hirai, Diogo F. C. Landim, Paola Zucchi

**Affiliations:** 1 Department of Ophthalmology and Visual Sciences, Escola Paulista de Medicina, Universidade Federal de São Paulo, São Paulo, SP, Brazil; 2 Discipline of Health Management and Economics, Escola Paulista de Medicina, Universidade Federal de São Paulo, São Paulo, SP, Brazil

**Keywords:** Glaucoma, Trabeculotomy, Laser therapy, Cost analysis, Health care cost Unified Health System, Brazil

## Abstract

**Purpose:**

To evaluate the economic impact of the following initial treatment scenarios for glaucoma on the Brazilian Public Health System (SUS): (1) traditional continuous instillation of hypotensive eye drops and (2) single session of selective laser trabeculoplasty.

**Methods:**

Economic impact was analyzed in three scenarios, from the least to the most conservative, for a hypothetical cohort of 5,000 individuals with open-angle glaucoma. Thereafter, projections were made on the basis of a glaucoma prevalence of 3% in the 2021 Brazilian population size.

**Results:**

All three scenarios demonstrated that selective laser trabeculoplasty exhibited a significantly lower economic impact than the eye drops on SUS over one and five years. Furthermore, the difference was more than United States Dollar 8 billion at five years when considering 3% of the Brazilian population aged >40 years in 2021.

**Conclusion:**

As the initial treatment for primary open-angle glaucoma, selective laser trabeculoplasty exhibited a lower economic impact on SUS than latanoprost and timolol maleate eye drop instillation in all the studied scenarios over one and five-year periods.

## INTRODUCTION

Glaucoma is an optic neuropathy that is characterized by a slow and progressive degeneration of the retinal ganglion cells (RGCs). This results in typical structural changes in the optic nerve head and retinal nerve fiber layer (RNFL) as well as corresponding defects in the visual field. RGCs are central nervous system neurons, with cell bodies and axons that are located in the inner retina. The axons organize themselves internally, forming bundles that constitute the RNFL. They converge at the optic disc, and form the optic nerve^(1)^.

According to the World Health Organization, glaucoma is the second leading cause of blindness worldwide and the leading cause of irreversible blindness^(2)^. Similar data has been found in the greater São Paulo area^(3)^. In a Brazilian study, the prevalence of primary open-angle glaucoma (POAG) in people aged >40 years old was 2.4% in whites and 3.8% in nonwhites^(4)^. Furthermore, its incidence appears to increase with age in all the studied populations^(1)^.

The clinical treatment of glaucoma classically begins with hypotensive eye drops, particularly prostaglandin analogs or beta-blockers, with the addition of other medications for adequate intraocular pressure (IOP) reduction. Each group of ocular hypotensive drug has a primary mechanism of action such as reducing aqueous humor production or increasing the uveoscleral or trabecular outflow. Long-term treatment with eye drops often results in side effects and requires good patient adherence^(5^,^6)^.

The introduction of laser (light amplification by stimulated emission of radiation) for the treatment of glaucoma in the latter half of the 20th century significantly impacted the management of various clinical conditions. In glaucoma, the commonly exploited laser effects are thermal, via argon laser, and ionizing, via neodymium-doped yttrium aluminum garnet (Nd:YAG) and diode lasers. Patients with open-angle glaucoma, the IOP can be reduced via laser trabeculoplasty. Selective laser trabeculoplasty (SLT) involves applying a frequency-doubled Nd:YAG laser directly to the trabecular meshwork. This leads to trabecular remodeling, which increases drainage via this route and reduces the IOP^(7^,^8)^.

Despite its temporary effect of approximately 36 months, initial glaucoma treatment with trabeculoplasty positively impacts a patient’s quality-of-life, reducing the need for IOP control with eye drops and the occurrence of side effects. Trabeculoplasty is an outpatient procedure with a good safety profile, and it can be repeated if needed. In the LiGHT Trial^(9)^, a multicenter randomized clinical trial that compared SLT with prostaglandin analog eye drops, approximately 350 participants were included in each group. Most of the patients had early glaucoma. The study’s findings revealed that initial laser treatment is safer, more cost-effective, equally or more effective, and less dependent on patient adherence to treatment than eye drop instillation. On average, in the group treated with trabeculoplasty, IOP control was achieved in 78% of the patients at the end of 36 months. Furthermore, 77% of these required only one laser treatment session^(9)^. Given the budgetary constraints, need for rational use of public resources, and adherence to best clinical practices, the cost of treatments offered by the Brazilian Public Health System (SUS) must be continuously evaluated. In Brazil, studies regarding the costs of glaucoma treatment are scarce. Guedes et al. evaluated the cost-effectiveness of clinical treatment (with eye drops) or laser treatment (unspecified trabeculoplasty) against observation, which is no longer an accepted line of management upon diagnosis of the disease. They concluded that both strategies were cost-effective, with a slight advantage with laser treatment. Furthermore, both alternatives significantly improved the patients’ quality-of-life^(10)^.

Determining the initial treatment for glaucoma with the least economic impact is imperative for SUS. Although SLT requires specific equipment and a qualified ophthalmologist to be performed, we hypothesize that the economic impact of SLT is lower than that of the traditional hypotensive eye drops. Therefore, in this study we aimed to evaluate the economic impact of the following two scenarios on SUS: (1) the traditional continuous clinical treatment with hypotensive eye drops (timolol maleate or latanoprost) and (2) single treatment with SLT.

## METHODS

Because the effectiveness of initial treatment with prostaglandin analog eye drops is similar to that with SLT, we used cost-minimization analysis (CMA) to evaluate economic feasibility. In CMA, the cost difference between alternative interventions that are assumed to produce equivalent results is calculated, with the interventions only differing in their incurred costs. When two strategies have the same therapeutic efficacy and health outcomes but different costs, the lower-cost strategy is preferred^(11)^.

An economic impact analysis was conducted according to the recommendations of the Brazilian Ministry of Health using data of individuals aged >40 years with glaucoma. The study’s sample size and characteristics were calculated on the basis of the prevalence estimates for the Brazilian population. The efficacy and safety of the two treatments are well established in the literature and are similar.

We assumed that the SUS would provide one of the following treatments to the entire target population for 48 months: latanoprost eye drops, timolol eye drops, or SLT.

### Target population

A hypothetical cohort of 5,000 individuals aged >40 years with POAG was adopted. Subsequently, to demonstrate the budgetary impact on public funds, this number was projected for the 2021 estimates of the Brazilian population by the Brazilian Institute of Geography and Statistics (IBGE). Finally, we considered the average prevalence of POAG in a population aged >40 years as 3.0% on the basis of international epidemiological studies and the national study by Sakata et al.^(4)^.

### Medication and procedure costs

#### Medication

Assuming that one bottle of latanoprost or timolol eye drops lasts 30 days when used in both eyes of an individual, its maximum consumer price was provided by the Drug Market Regulation Chamber. The average acquisition cost was also determined by accessing the Price Panel of the Brazilian Ministry of Economy. This is because purchasing in larger quantities and at lower costs is mandatory in the federal public service. The cost of eye drops in the Brazilian Ministry of Health’s Glaucoma Program were not used because it also includes the price of consultation and some tests.

#### Laser procedure

The cost of SLT was determined from the management system of the SUS Table of procedures, medications, and medical supplies (SIGTAP). The total cost of two procedures (one for each eye) during the same period, according to efficacy studies, was considered^(9)^. Additionally, expenses (free on board [FOB] price in Miami, USA) for the acquisition (single), installation (single), operation (annual), and maintenance (annual) of the laser equipment, as well as its depreciation, were added to the cost of scenario 3.

The average depreciation rate for equipment is 10% per year, with a useful life of 10 years. Depreciation was calculated using the following formula: (AC - RV) x r; where “AC” is the acquisition cost of the asset, “RV” is its residual value, and “r” is the corresponding depreciation rate.

#### Sensitivity analysis

The cost difference of the alternative scenario (SLT) was calculated in comparison to the two reference scenarios (use of latanoprost eye drops or timolol maleate eye drops) over oneand five-year analytical horizons^(12^,^13)^.

### Scenarios

In the first scenario (Table 1), a nonconservative analysis was adopted. This was the best scenario for eye drops because of the constant values from the Ministry of Economy Price Panel and the following usage distribution: 70%, timolol maleate (lower cost) and 30%, latanoprost (higher cost between the two).

**Table 1:**
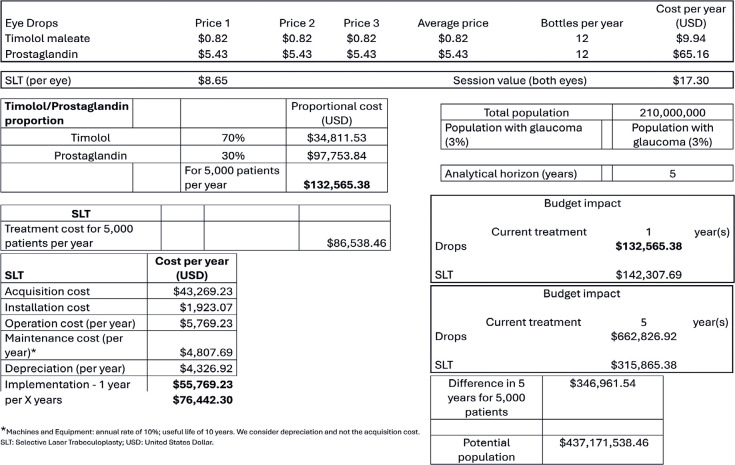
Economic impact on the Brazilian public health system using the non-conservative analysis (best case scenario for eye drop type distribution)

In the second scenario (Table 2), a conservative analysis was adopted. This was the worst scenario for eye drops because of the constant values from the Ministry of Economy Price Panel and the following usage distribution: 70%, latanoprost (higher cost) and 30, timolol (lower cost between the two).

**Table 2:**
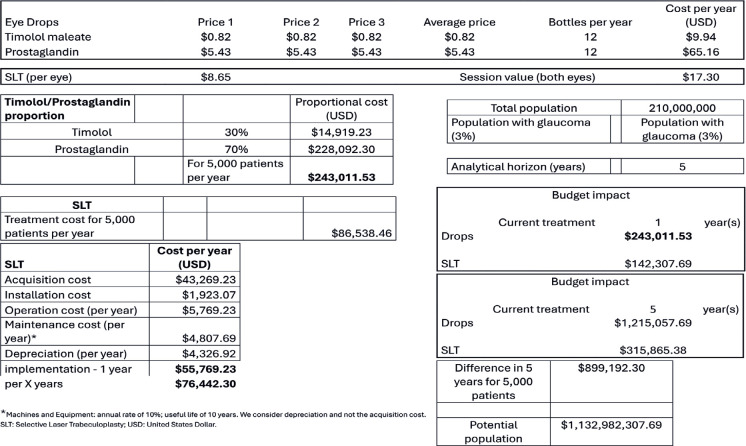
Economic impact on the Brazilian public health system using the conservative analysis (worst case scenario for eye drop type distribution)

In the third scenario (Table 3), a super conservative analysis was adopted. This was the worst scenario for eye drops with the maximum consumer price because of the constant values from the Drug Market Regulation Chamber (the average of the two lowest values and the highest value due to the Brazilian Tax on the Circulation of Goods and Services - ICMS) and the following usage distribution: 70%, latanoprost (higher cost) and 30% timolol (lower cost between the two).

**Table 3:**
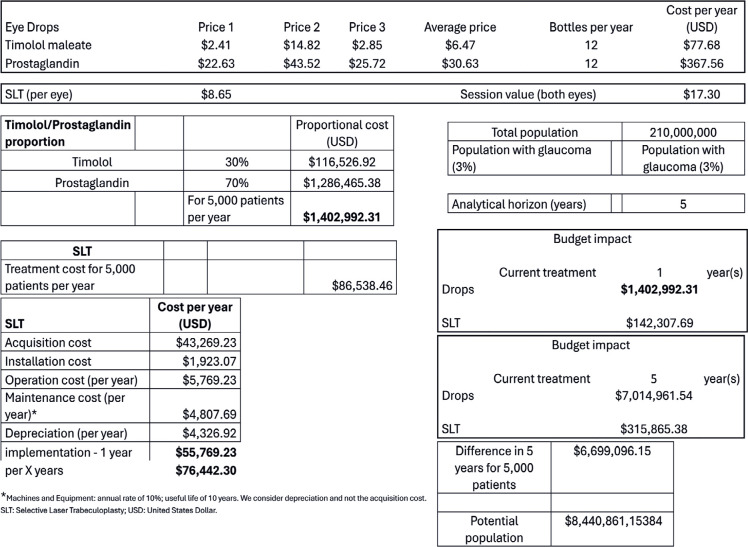
Economic impact on the Brazilian public health system using the conservative analysis 2 (worst case scenario for eye drop type distribution + maximum consumer price)

## RESULTS

The prices of medications from the latest government purchases (Ministry of Economy Price Panel website: https://paineldeprecos.planejamento.gov.br/; accessed on 06/29/2022), the maximum consumer price (Drug Market Regulation Chamber - CMED - National Health Surveillance Agency; updated on 06/29/2022), and the SUS SIGTAP fore SLT (code 04.05.05.012) were considered. The prices in these databases were converted from Brazilian Real (BRL) to United States Dollar (USD) at an exchange rate of 5.2 (Central Bank of Brazil, 06/29/2022). An FOB price (in Miami, USA) of approximately USD 43,269.23 was considered for the laser equipment.

In the first scenario, the economic impact of the treatments was similar at one year and lower for SLT at five years. The difference amounted to USD 346,961.54 among 5,000 individuals, which reached USD 437,171,538.46 when the national population prevalence was considered.

In the second scenario, the economic impact of the treatments was advantageous for SLT at one year (>500,000 reals for 5,000 people) and much lower for laser at five years. The difference was USD 899,192.30 for 5,000 individuals, which reached USD 1,132,982,307.69 when the national disease prevalence was considered.

In the third scenario, the economic impact of the treatments was extremely advantageous for SLT at one year and much lower for laser at five years. The difference was USD 6,699,096.15 for 5,000 individuals, which reached a substantial amount of USD 8,440,861,153.84 when considering the national glaucoma prevalence.

## DISCUSSION

The treatment of patients diagnosed with POAG has traditionally begun with the continuous daily instillation of hypotensive eye drops. According to medical society protocols and Brazilian Ministry of Health agencies, first-line treatments include beta-blockers (timolol maleate 0.5%) and prostaglandin analogs (latanoprost, bimatoprost, travoprost, and tafluprost). The beta-blockers are instilled twice a day and cost lesser than prostaglandin analogs, which are instilled once a day at night^(14)^. The chronic use of eye drops poses challenges and limitations, such as higher cost, adherence to treatment, potential reduction in the quality-of-life, and local and systemic side effects from both the active ingredient and preservatives^(5^,^6)^.

SLT is a one-time procedure with a therapeutic result that is similar to that of the continuous use of a first-line hypotensive eye drop^(9)^. However, it depends on the laser equipment, which has acquisition and maintenance costs as well as depreciation. Furthermore, its use requires a trained ophthalmologist.

The three analyzed scenarios demonstrated that SLT exhibits a significantly lower economic impact on SUS over 1-5 years. The use of laser exhibited a favorable difference of more than USD 8 billion over five years when considering 3% of the Brazilian population in 2021. Thus, adoption of SLT as the initial therapy for POAG in Brazil would ensure an economic advantage. Furthermore, it would allow for the improvement of reimbursements for the procedure, in which an old class of laser (argon) is used when the selective YAG laser has already used. This accounts for the current nomenclature of SLT.

In conclusion, as the initial treatment for POAG, SLT exhibited a lower economic impact on SUS than latanoprost and timolol maleate eye drops in all the studied scenarios over oneand five-year periods.
